# The GLP‐1/GIP dual‐receptor agonist DA5‐CH inhibits the NF‐κB inflammatory pathway in the MPTP mouse model of Parkinson's disease more effectively than the GLP‐1 single‐receptor agonist NLY01

**DOI:** 10.1002/brb3.2231

**Published:** 2021-06-14

**Authors:** MiaoJun Lv, GuoFang Xue, HuiFeng Cheng, PengFei Meng, Xia Lian, Christian Hölscher, DongFang Li

**Affiliations:** ^1^ Second Hospital, Neurology Department Shanxi Medical University Taiyuan Shanxi Province People's Republic of China; ^2^ Research and Experimental Center Henan University of Chinese Medicine Zhengzhou Henan Province People's Republic of China

**Keywords:** apoptosis, autophagy, BBB, growth factor, insulin, oxidative stress

## Abstract

The GLP‐1 receptor agonist exendin‐4 has recently shown good effects in a phase II clinical trial in Parkinson's disease (PD) patients. Here, a comparison of the new GLP‐1/GIP dual receptor agonist DA5‐CH and NLY01, a 40 kDa pegylated form of exendin‐4, on motor impairments and reducing inflammation in the 1‐methyl‐4‐phenyl‐1,2,3,6‐tetrahydropyridine (MPTP) PD mouse model is provided. The drug groups received either DA5‐CH or NLY01 (25 nmol/kg) i.p. after daily MPTP intraperitoneal injection. Both drugs showed improvements in motor activity, open field experiments, rotarod tests, and gait analysis, but DA5‐CH was more potent. Tyrosine hydroxylase expression in dopaminergic neurons was much reduced by MPTP and improved by DA5‐CH, while NLY01 showed weak effects. When analyzing levels of α‐synuclein (α‐Syn), DA5‐CH reduced levels effectively while NLY01 had no effect. When measuring the levels of the inflammation markers Toll‐like receptor 4 (TLR4), specific markers of microglia activation (Iba‐1), the marker of astrocyte activation glial fibrillary acidic protein (GFAP), nuclear factor‐κB (NF‐κB), tumor necrosis factor (TNF‐α), and transforming growth factor β1 (TGF‐β1), DA5‐CH was very effective in reducing the chronic inflammation response, while NLY01 did not show significant effects. Levels of key growth factors such as Glial cell‐derived neurotrophic factor (GDNF) and Brain‐derived neurotrophic factor (BDNF) were much reduced by MPTP, and DA5‐CH was able to normalize levels in the brain, while NLY01 showed little effect. The levels of pro‐inflammatory cytokines (IL‐6 and IL‐Iβ) were much reduced by DA5‐CH, too, while NLY01 showed no effect. In a separate experiment, we tested the ability of the two drugs to cross the blood‐brain barrier. After injecting fluorescin‐labelled peptides peripherally, the fluorescence in brain tissue was measured. It was found that the pegylated NLY01 peptide did not cross the BBB in meaningful quantities while exendin‐4 and the dual agonist DA5‐CH did. The results show that DA5‐CH shows promise as a therapeutic drug for PD.

## Graphical abstract

GLP‐1 analogues have been shown in clinical trials to reduce Parkinson disease progression. The study compares two drugs from this group in a mouse model of PD. The drug NLY01 which is currently tested in a clinical trial in PD patients showed only limited protective effects, compared to a novel drug that can cross the blood‐brain barrier.

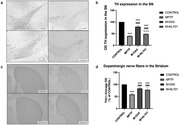

## 1 INTRODUCTION

Parkinson's disease (PD) is one of the most common neurodegenerative diseases that occur in middle‐aged and elderly people. With the advent of an aging society in developed countries, its morbidity, prevalence, and mortality have increased significantly, becoming an important burden of public health. The current treatment plan for dopamine or dopamine precursors to improve the symptoms of PD does not significantly prevent disease progression and is accompanied by some side effects and complications (Aviles‐Olmos et al., 2013). The main pathological feature of PD is that the death of dopaminergic nerve cells in the substantia nigra compact part of the midbrain. When the cell loss reduction reaches more than 70%, a series of motor dysfunction symptoms such as resting tremor, muscle stiffness, and slow movement will occur. However, the specific pathogenesis and development mechanisms are still unclear. Mitochondrial dysfunction, oxidative stress, neuroinflammation, apoptosis, necrosis, and autophagic death may all be involved in the death of dopaminergic neurons. Research on the prevention and treatment of Parkinson's disease is still ongoing.

In recent years, research on immune‐related regulatory mechanisms of the nervous system has become a focus of research. Studies have found that the development of many central chronic degenerative diseases are accompanied by a chronic immune inflammatory reaction (Caggiu et al., 2019). Chronic inflammation can damage the repair pathways of the brain, and glial cells are important in this process by releasing pro‐inflammatory cytokines and nitric oxide (Tansey & Goldberg, 2010). When infection or other nerve damage occurs, the innate immune system, including astrocytes and microglia, is activated by toll‐like receptors (TLR). Among them, TLR4 is highly expressed on the membrane of microglia, and is highly involved in neuroinflammation during central nervous system injury, and has a regulatory effect on nuclear factor NF‐κB (p65) (Ferrari & Tarelli, 2011; Perry et al., 2007; Togashi et al., 1997). The expression of NF‐κB participates in many physiological reactions, including immune inflammation, acute inflammation, oxidative stress, cell adhesion, differentiation, and apoptosis (Togashi et al., 1997). In PD brain, α‐Syn is involved in inflammatory activation and is considered as a key driver of PD (Bengoa‐Vergniory et al., 2017).

Studies have shown that many neurological diseases are associated with T2DM (Type 2 Diabetes Mellitus). Both of them gradually increase with age and show a chronic progression. The risk of Parkinson's disease in T2DM patients seems to be higher and disease progression is faster (Cheong et al., 2020; Miyake et al., 2010). The risk of developing PD increases with the duration of fasting blood glucose/DM impairment, and the severity gradually increases (Foltynie & Athauda, 2020; Xu et al., 2011). Glucagon‐like peptide 1 (GLP‐1) is a peptide hormone with growth‐factor‐like properties, GLP‐1 is produced in several tissues including the brain. GLP‐1 released into the bloodstream can cross the blood‐brain barrier and enter the central nervous system to play a role (Hölscher, 2018; Kastin et al., 2002). GLP‐1 receptor (GLP‐1R) is widely expressed in the central nervous system, including hippocampus, neocortex, hypothalamus, and cerebellum, especially in midbrain and striatum (Darsalia et al., 2012; 2013; During et al., 2003; Graham et al., 2020; Hamilton & Holscher, 2009; Lee et al., 2011; Li et al., 2009; Merchenthaler et al., 1999; Teramoto et al., 2011). GLP‐1R agonists have shown consistent neuroprotective effects in a range of cell culture and animal models of disease (Hölscher, 2018). Studies have shown that exendin‐4 pretreatment can reduce MPTP‐mediated dopaminergic neurodegeneration, and can cross the blood‐brain barrier and reduce brain inflammation by inhibiting the recruitment and activation of glial cells (Aviles‐Olmos et al., 2013; Zhang et al., 2020). Exendin‐4 has a protective effect on central system diseases, and was recently found to have a beneficial effect on motor function in a randomized placebo‐controlled phase II trial for the treatment of PD (Athauda et al., 2017, 2019). On the basis of this, a pegylated version of exendin‐4 is being tested as a potential drug treatment for PD. This drug named NLY01 has extended half life in the blood stream and has a 40 kDa pegylation added to the exendin‐4 peptide (Kim et al., 2012; Yun et al., 2018). NLY01 has been shown to have protective effects in different animal models of inflammation and PD (Yun et al., 2018). Currently, NLY01 is being tested in a phase II clinical trial in PD patients (NCT04154072).

Glucose‐dependent intestinal insulin‐releasing peptide (GIP) is the “sister” incretin hormone of GLP‐1. GIP analogs originally designed for the treatment of diabetes showed good protection in animal models of AD and PD (Zhang & Holscher, 2020). Our previous studies have proved that the new long‐acting GIP analog D‐Ala2‐GIP‐Glu‐PAL has a neuroprotective effect on acute and chronic MPTP mouse models (Li et al., 2016, 2017).

Nowadays, there are new GLP‐1/GIP dual receptor agonists, which are more effective in diabetic patients, better than a single GLP‐1 or GIP, which can fully exert the pharmacological effects of the two while reducing related side effects (Finan et al., 2013). In direct comparisons, the novel GLP‐1/GIP dual receptor agonists are more effective than single GLP‐1 or GIP analogues in mouse models of PD (Feng et al., 2018; Zhang et al., 2020). Crossing the blood‐brain barrier (BBB) is an important property of drugs that can reduce neurodegenerative disorders in the CNS (Scherrmann, 2002). We have developed two novel dual GLP‐1/GIP receptor agonists that can cross the BBB better due to CPD modifications (Feng et al., 2018; Hölscher, 2018; Li et al., 2020; Salameh et al., 2020; Zhang et al., 2020). In direct comparisons, they are superior in entering the brain compared to other GLP‐1 or GIP or dual GLP‐1/GIP receptor agonists (Li et al., 2020; Salameh et al., 2020; Zhang et al., 2020). In contrast, the pegylated NLY01 version of exendin‐4 has poor BBB penetration capabilities (Yun et al., 2018) (see Figure S2). In order to directly compare the dual GLP‐1/GIP receptor agonist DA5‐CH that has shown superior neuroprotective effects in the MPTP mouse model of MPTP with the NLY01, we compared their effects at the same dose and measured the effects on motor symptoms and the chronic inflammatory response in the brain, among other key biomarkers.

## 2 MATERIALS AND METHODS

### 2.1 Laboratory animals, reagents, and instruments

#### 2.1.1 Experimental animals

C57BL6/J male mice were used, 8 weeks old, 22–25 g weight, SPF rearing conditions, purchased from the Experimental Animal Center of Shanxi Medical University. They were reared under constant temperature (22±3)°C, constant humidity (50%−55%), 12:12 h light and dark cycle conditions, four mice in each cage, ensure adequate diet, drinking water, and proper ventilation, fasting for 8 hours before detecting fasting tail vein blood glucose. Before entering the model building, each mouse was given an adaptation period of about 14 days to reduce experimental errors caused by environmental inadaptability. All animal experiment procedures in this study were approved by the Ethics Committee of Shanxi Medical University and implemented in accordance with the National Institutes of Health (National Institutes of Health, NIH) guidelines (NIH publication, No. 85‐83, revised in 1985).

#### 2.1.2 Experimental reagents

DA5‐CH powder and NLY01 were obtained from Chinapeptides (Shanghai, China). The purity was 95% as detected and determined by reversed‐phase high‐performance liquid chromatography and flight mass spectrometer.

The DA5‐CH peptide aa sequence is YXEGTFTSDYSIYLDKQAAXEFVNWLLAGGPSSGAPPPSKRRQRRKKRGY‐NH2 (X = aminoisobutyric acid) (Feng et al., 2018).

The peptide aa sequence of NLY01 is HGEGTFTSDLSKQMEEEAVRLFIETLKNGGPSSGAPPPSC‐PEG (Yun et al., 2018).

The drugs were dissolved in 0.9% normal saline at a concentration of 25 nmol/kg/d, i.p. MPTP was purchased from Sigma (USA), with 4% paraformaldehyde (Boster Institute of Biotechnology, Wuhan, China). Ethylenediamine tetra‐acetic acid (EDTA) antigen retrieval solution, diaminobenzidine (DAB) color developing solution, horseradish peroxidase labeled goat anti‐rabbit IgG (ZSGB‐B IO, China); β‐actin (1:5000; Bioworld, USA), GAPDH (1:5000; Bioworld, USA), TH (1:200, Abcam, UK), α‐Syn (1:2000, Abcam, UK), GFAP (1:10000; Abcam, UK), rabbit anti‐IBA‐1 (1:100; Bioworld, USA), rabbit anti‐NFκB‐p65 (1:500, Bioworld, USA), TNF‐α (1:500; Bioworld, USA)), rabbit anti‐TGF‐β1 (1:500; Bioworld, USA), rabbit anti‐TLR4 (1:100, ABclonal, Wuhan, China), rabbit anti‐GDNF (5μg/ml; Abcam, UK), rabbit anti‐BDNF (1 :50; Bioworld, USA), and secondary peroxidase‐conjugated antibody (1:5000; Bioworld, USA). Phenylmethanesulfonyl fluoride (PMSF), radioimmune precipitation assay (RIPA) lysate, sodium dodecyl sulfate polyacrylamide gel electrophoresis (SDS‐PAGE) Protein loading buffer 5X, bicinchoninic acid (BCA) protein quantification kit, goat anti‐rabbit IgG conjugated with peroxidase, and ultra‐sensitive ECL chemiluminescence ready‐to‐use substrate (Boster, China).

#### 2.1.3 Experimental instruments

Blood glucose meter (Corfu, China), electronic balance, fatigue rotating rod meter (type: ZN17‐YLS‐4C), flat panel gait analyzer (CleverSys Inc., USA); automatic biological tissue dehydrator TP1020, manual rotary type Microtome RM2235, spread dryer HI1220, microscope DM1000 (Leica, Germany); high‐speed refrigerated centrifuge 5810 (Eppendorf, Germany); micro sampler, microplate reader Multi Skan MK3 (Thermo Scientific, USA); protein Electrophoresis instrument, protein transfer membrane instrument, gel imaging system (Bio‐Rad, USA).

#### 2.1.4 Blood‐brain barrier penetration study

We tested the peptides exendin‐4, NLY01, and DA5‐CH. Three‐month‐old C57BL6 mice were injected with fluorescein‐labelled peptide at 50 nmol/kg i.p., *n* = 6 per group. This dose has previously shown good effects in BBB studies of DA5‐CH and similar peptide drugs (Li et al., 2020; Salameh et al., 2020; Zhang et al., 2020). Two hours later, animals were anaesthetized with Dolethal pentobarbital (BAYER) at 50 mg/kg and were transcardially perfused with 20 ml phosphate buffered saline (PBS, pH7.4), followed by approximately 20 ml 4% paraformaldehyde (PFA, pH 7.4). Brains were post‐fixed in PFA for 24 h in 20% sucrose solution and sectioned on a Leica cryostat at 40 μm, and fluorescence was quantified on a Zeiss Axio Scope A1 microscope under 488 nm illumination. Images were taken with a Sony ICX 267 digital camera at 525 nm. One image per brain was taken at a random location in the cortex. The image size was 250 × 250 μm. Fluorescence was quantified using Image‐Pro Plus 6.0 software (Media Cybernetics, Inc., USA). For details see Li et al. (2020) and Zhang et al. (2020).

#### 2.1.5 MPTP treatment

MPTP was dissolved in physiological saline, packaged separately, and stored in a refrigerator at −80°C. After the mice were weighed, and were injected intraperitoneally at 25 mg/kg each time at 8 am every day for 7 consecutive days. The success criteria for inducing a PD‐like state were: continuous limb tremor, stiffness of the limbs, erect hair, frequent swallowing activities, restricted activities, slow movement, arched back, and tail stiffness after the injection of the mice on the 6th to 7th days. For a timeline of the experiment see Figure 2.

#### 2.1.6 Experiment grouping

After two weeks of acclimatization to the suitable environment in the animal room, 72 male C57BLs were randomly divided into 4 groups, each of which was *n* = 18: A) control group: Normal saline (0.1 ml/day, once, i.p., 7 days); B) MPTP model group: MPTP (25 mg/kg, once, ip, 7 days) + normal saline (0.1 ml/day, once, i.p., 7 days); C) DA5‐CH+MPTP group: MPTP (25 mg/kg, once, i.p., 7 days) + DA5‐CH (25 nmol/kg/day, once, i.p., 7 days); D) NLY01+MPTP: MPTP (25 mg/kg/2 h, 1 time, i.p., 7 days) + NLY01 (25 nmol/kg, 1 time, i.p., 7 days). The dose of DA5‐CH was the same as used in previous studies (Li et al., 2020; Zhang et al., 2020).

#### 2.1.7 Blood glucose and weight testing

Ten mice in each group were selected for blood glucose testing (collecting blood from the tail vein, using a blood glucose meter, and recording the value) before injecting the drug on the first day. The weight of these 10 mice was taken on the first and seventh day.

### 2.2 Behavioral testing

#### 2.2.1 Rotarod test

This tests the motor ability of a mouse to coordinate motor activity and balance on a rotating rod. The test instrument (model YLS‐4C, Shandong Medical University, China) consists of a rotating shaft and five independent compartments to test five mice at the same time. Before drug administration, all mice were given preparatory training for three consecutive days. During the test, the mouse was placed on the rotating rod, and the rotating rod was accelerated from 0 to 20 rpm, and the acceleration rotation time was set to 180 s. On the 7th day, a formal test was performed 1 h after the injection, and two tests were performed at an interval of half an hour. The total time was set to 180 s, and the speed of the rotating rod was increased from 0 to 30 rpm. During the test, the staying time of each group of experimental mice on the rotating rod was recorded, and the final results were averaged for three times.

#### 2.2.2 Open field test

In this study, the device consists of a circular open field (35 cm in diameter; 40 cm in height) and uses a computer tracking system (Etho Vision XT software, Noldus Information Technology, Wageningen, Netherlands) to record animal behavior. After adapting to the environment the night before, each mouse was placed in the center of the open field, and its movement activities were recorded in the next 10 minutes and analyzed by a computer tracking system. After each mouse was tested, the area was wiped with 75% ethanol to prevent errors caused by olfactory response. The open field experiment was tested on the 7th day by a person who did not know the groups’ identity.

#### 2.2.3 Gait analysis test

In recent years, the gait analysis method has become an important method for evaluating the severity of PD, which makes up for the various shortcomings of some methods of behavioral testing, and meets the growing demand for basic research. C57BL/6 mice freely pass through the 115 cm long and 6.5 cm wide glass runway on the gait analyzer in the dark environment 2–3 times in 20 seconds (2000 frames), during which one of the lengths is set The paw prints of the (30 cm) glass track were collected by BcamCapture software and analyzed by RunwayScan software. The mice adapt to the dark environment for one night. Before the test, each mouse is placed on the track and allowed to explore the habit track to ensure that it must pass through the set 30 cm length glass track in one direction continuously and without pause. After the RunwayScan3.0 analysis system has identified and marked each footprint, it will automatically generate a series of gait parameters, including stride length and etc. Each foot can collect at least 11 consecutive footprints for its parameters statistics. Before the gait test, we weighed the mice in each group, and all mice must weighed close to 20‐30 g. During the experiment, the data of mice that were overweight or underweight, and those that stopped, turned or stagnated within the set length of 30 cm, were not included in the statistical analysis.

### 2.3 Specimen preparation and testing

#### 2.3.1 Separation and fixation of mouse brain tissue

After behavioral evaluation, each group of mice was divided into two parts. The first part is the immunohistochemical staining. The mice (*n* = 6 in each subgroup) were anesthetized with 10% chloral hydrate (5 ml/kg, intraperitoneal injection), followed by 100 ml of normal saline and 100 ml of 4% PFA. A solution of paraformaldehyde (PFA) is used for cardiac perfusion to replace blood and fix tissue. The mice were quickly decapitated, brains were removed and placed in 4% PFA solution, and fixed for another 12 hours. Gradient dehydration was carried out using an automatic biological tissue dehydrator, and paraffin embedding. Western blot: the mice (*n* = 6 in each subgroup) were anesthetized with 10% chloral hydrate, and the heart was perfused with 200 ml of normal saline. The whole brain tissue was quickly dissected and collected on ice. The substantia nigra and the striatum tissue was isolated and stored at −80°C.

#### 2.3.2 Western blotting

After lysing the brain tissue of each group of mice with PMSF RIPA lysate and extracting the total protein, the protein concentration was quantified by BCA method. After adjusting the protein concentration with RIPA Lysis Buffer (Beyotime, China), the same 20 mg protein was used for electrophoresis. The protein was separated by SDS‐PAGE gel electrophoresis (concentrated gel constant pressure 80 V + separation gel constant pressure 100 V). Then, the protein was transferred to the PVDF membrane with the following rabbit anti‐mouse primary antibodies at 4°C overnight:β‐actin, GAPDH, TH, α‐Syn, GFAP, rabbit anti‐IBA‐1, rabbit anti‐NFκB‐p65, TNF‐α, and rabbit anti‐TGF‐β1. On the second day, the membrane was incubated with the secondary antibody at room temperature for 1 h. After washing with TBST, ECL was used to enhance chemiluminescence imaging (Boster, China). After the film was scanned, β‐actin or GAPDH was used as the internal reference to analyze the relative absorbance value of the target band with the Image Lab 3.0 software gel image processing system.

#### 2.3.3 Immunohistochemical analysis

Using a semi‐automated microtome (Leica, Wetzlar, Germany), the range of the striatum area from the bregma from 1.54  to 0.10 mm, and the range of the substantia nigra compact from 2.92  to 3.40 mm from the bregma were taken from each group of mice. Coronal slices of 4 μm thick serial were cut. The number of positive cells were calculated, and the average cell count of each animal for statistical analysis was used. Criteria for positive judgment: the cytoplasm is brown under the light microscope, and the nucleus is light blue or purple blue. This study used TH, TLR4, GFAP, IBA‐1, TNF‐α, NF‐κB‐p65, GDNF, BDNF, TGF‐β1 and secondary peroxidase binding antibodies. Magnification was x 400. Image‐Pro Plus 6.0 software was used to count cells.

#### 2.3.4 Analysis of cytokine levels

Mouse midbrain tissue was homogenized with trypsin to make a homogenate. Instructions for the enzyme‐linked immunosorbent assay kit (Boster, China) to measure IL‐1β, IL‐6 and IL‐10 were followed.

#### 2.3.5 Statistical methods

Statistical software SPSS23.0 was used for analysis. Measurement data are expressed as mean ± standard deviation, comparison between two groups was by *t*‐test, between three or more by one‐way analysis of variance (ANOVA) with post‐hoc tests. Weight and glucose levels were analyzed by repeated measurement of the two‐way difference using the Prism 7 stats program (Graphpad software, USA). *p* < .05 was considered statistically significant.

## 3 RESULTS

### 3.1 BBB penetration of drugs

**FIGURE 1:**
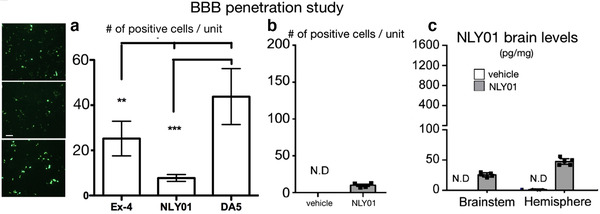
Analysis of BBB penetration by injecting fluorescence‐labelled peptides peripherally and analysing brain sections. (a) comparing DA5‐CH with exendin‐4 and NLY01. *N* = 6 per group. Sample images are shown, top: ex‐4, middle: NLY01, bottom: DA5. Scale bar = 10 μm. (b) results using the same technique as published in Yun et al. (2018), showing the same results as presented in our study. (c) quantification of NLY01 as published in (Yun et al., 2018) (redrawn Figure 2 B,C, supplementary file). *** = *p*<.001; ** = *p*<.01. N.D. = not detected

When comparing the number of positive fluorescin cells in brain tissue, an overall one‐way ANOVA found a difference between groups (*p* = .001). Post‐hoc tests revealed difference between groups. DA5‐CH had the highest numbers per micrograph (*p* < .001 compared with NLY01, *p* < .01 compared with exendin‐4). Exendin‐4 had higher numbers than NLY01 (*p* = .01) (see Figure 1(a)). For comparison, results from a previous study have been added (Figure 1(b) and 1(c)). *N* = 6 per group.

**FIGURE 2:**
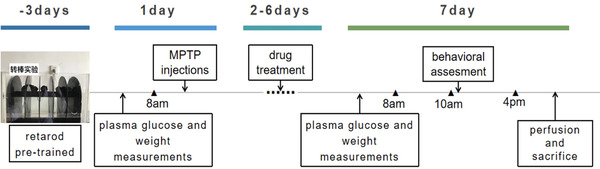
Schematic diagram of the experimental procedure

### 3.2 Blood glucose and weight monitoring

**FIGURE 3:**
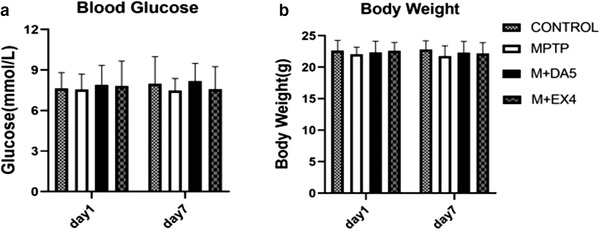
Analysis of blood glucose level and body weight of mice measured at the same point of tim of fasting in the early morning on day 1 and day 7. (a) No statistical difference was found when comparing the blood glucose levels of each group on the 1st and 7th day using the two‐factor repeated measurement analysis of variance method (time DF = 1, F = 0.089, *p* > .05; Group degrees of freedom groups DF = 3, F = 0.325, *p* > .05; interaction degrees of freedom interaction DF = 3, F = 0.283, *p* > .05). (b) There was no statistical difference when comparing the weight in each group on the 1st and 7th day by the two‐factor repeated determination analysis of variance method (time DF = 1, F = 0.156, *p* > .05; groups DF = 3, F = 0.873, *p* > .05; interaction DF = 3, F = 0.141, *p* > .05). *n* = 10 per group

When analyzing blood glucose levels and body weight on day 1 and day 7, two‐way repeated‐measure ANOVA found no difference between the groups. The results showed that the two groups of drugs had no effect on the body weight or blood sugar of the experimental mice (see Figure 3).

### 3.3 Motor activity of MPTP‐treated mice

#### 3.3.1 Rotarod test

**FIGURE 4:**
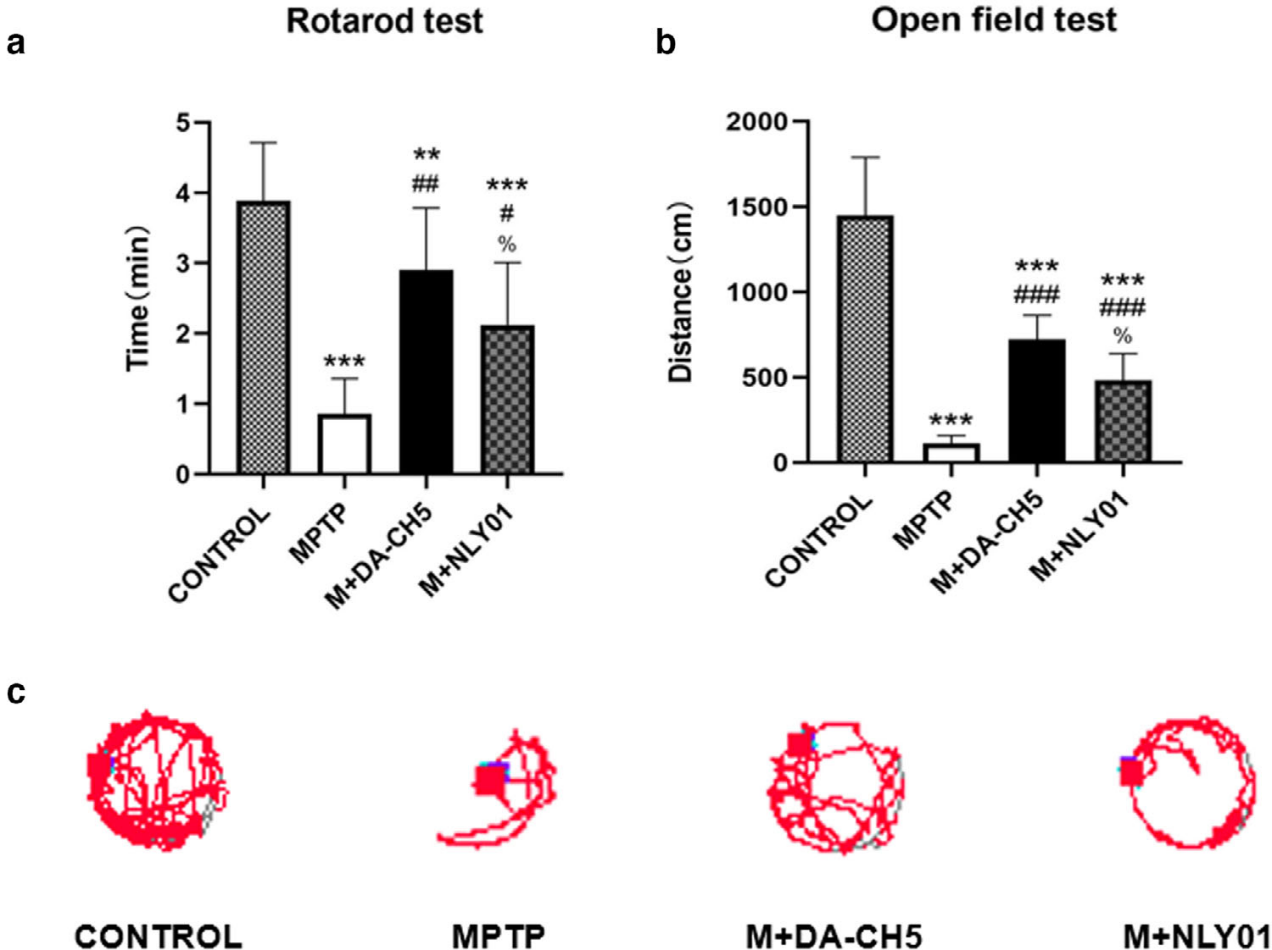
Muscle strength and space exploration of mice. (a) The results of the rotarod experiment showed that compared with the control group, the time spent on the rotating rod of the MPTP group was significantly reduced (*p*<.0001) compared with the MPTP group, the DA5‐CH group and the NLY01 group. The staying time on the rotating rod increased significantly (*p* < .01), and the DA5‐CH group had a more significant increase in the staying time than the NLY01 group (*p* < .05). (b) The results of the open field experiment showed that compared with the control group, the total moving distance of the mice in the MPTP group was significantly reduced (*p*<.0001); compared with the MPTP group, the total of the mice in the DA5‐CH group and the NLY01 group. Both the moving distance and the average movement speed values increased significantly (*p*<.0001), and the DA5‐CH group increased more significantly than the NLY01 group (*p*<.05). (c) Open field test trajectory samples of each group. *** = *p*<.0001 compared with the control group; ** = *p*<.01 compared with the control group; ### = *p*<.0001 compared with the MPTP group; ## = *p*<.01 compared with the MPTP group; # = *p*<.05 compared with the MPTP group; % = *p* < .05 compared with M+DA5‐CH group. *n* = 10 per group

A one‐way ANOVA found that there were differences between all groups, and the LSD comparison test between groups found that the differences between groups were significant. Compared with the MPTP group, both drugs restored the muscle strength and exercise performance of the model mice (*p* < .01), and DA5‐CH had a better effect (*p* < .05). See Figure 4(a).

#### 3.3.2 Open field test

A one‐way ANOVA found that there were differences between all groups, and the LSD comparison test between groups found that the differences between groups were significant. Compared with the MPTP group, both drugs restored the space exploration ability of the model mice in the new environment, the total movement distance of the mice was significantly increased (*p*<. 0001), and DA5‐CH had a better effect (*p*< . 0001) compared with NLY01. See Figure 4(b).

#### 3.3.3 Gait test

**FIGURE 5:**
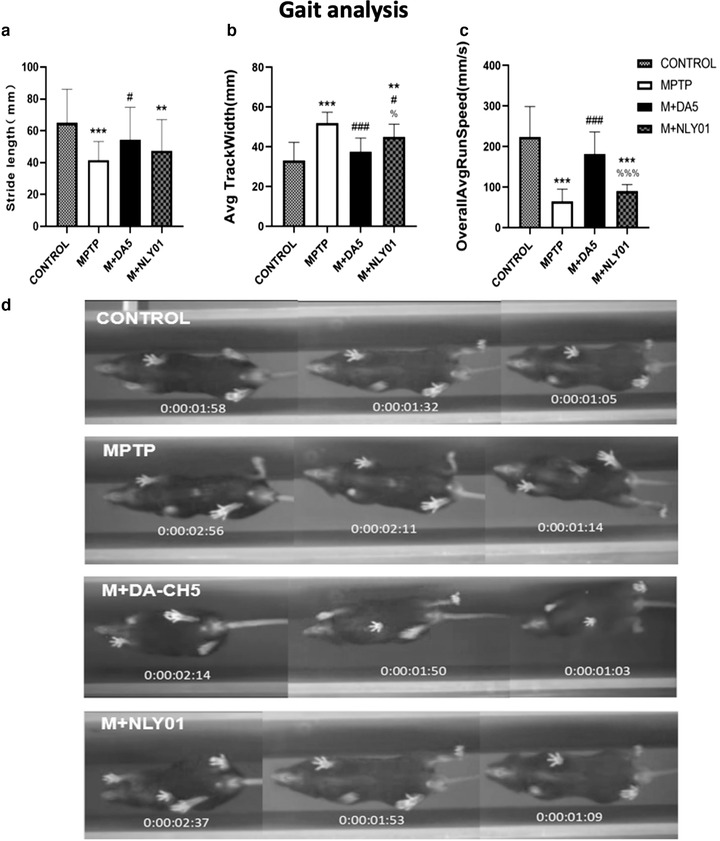
Walking gait analysis. (a) Compared with the MPTP group, the DA5‐CH group showed significant improvements in step length (*p* < .05), while the NLY01 group showed no statistically significant changes (*p* > .05). (b) Compared with the MPTP group, the DA5‐CH group showed a greater improvement (*p*<.0001) than the NLY01 group (*p*<.01). (c) Compared with the MPTP group, the DA5‐CH group had a significant improvement on the average speed of mice (*p* < .0001), and the average speed of mice in the NLY01 group was not statistically significant (*p* > .05). (d) Sample tracks of the gait analysis as captured by the digital video system

A one‐way ANOVA found that there were differences between all groups, and the LSD comparison test between groups found that the differences between groups were significant. Compared with the control group, the step length, stride width, and average speed of the model group had significant changes (*p* < .0001), indicating that the modeling drug had an effective effect and the modeling was successful. Compared with the MPTP group, DA5‐CH significantly restored the gait behavior of MPTP mice, significantly improving the stride width and average speed of the mice (*p* < .0001), and had statistical significance in improving the step length (*p* < .05), while NLY01 only improved the stride width (*p* < .05). See Figure 5.

### 3.4 Loss of dopamine neurons and the accumulation of α‐syn in the brain

In order to detect the effects of the two drugs, this subject detected and analyzed the levels of TH, a key enzyme of dopamine neurons, and the level of α‐syn, an important protein related to the pathogenesis of PD.

#### Western blot assay

**FIGURE 6:**
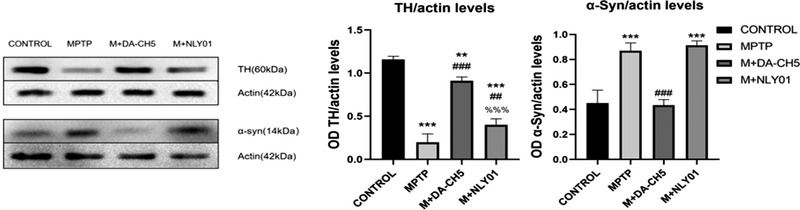
(a) Expression of TH and α‐syn in brain tissues, sample western blots. (b) Compared with the MPTP group, the TH levels of the DA5‐CH group and the NLY01 group increased significantly (*p*<.0001, *p*<.001), with DA5‐CH being more effective than NLY01 (*p*<.0001). (c) Compared with the control group, the α‐syn expression level of mice in the MPTP group was significantly increased (*p* < .0001); compared with the MPTP group, the α‐syn expression level of the mice in the DA5‐CH group and the NLY01 intervention group was significantly decreased (*p* < .0001), the DA5‐CH group had a more significant decrease in α‐syn expression level than the NLY01 intervention group (*p* < .0001). *** = *p*<.0001 compared with the control group; ** = *p*<.01 compared with the control group; ### = *p*<.0001 compared with the MPTP group; ## = *p*<.01 compared with the MPTP group; %%% = *p*<.0001 compared with the M+DA5‐CH group; *n* = 3 per group

One‐way ANOVA found that there were differences between all groups, and the LSD comparison test between groups found that the differences between groups were significant. In the midbrain tissue: compared with the MPTP group, both drugs restored the expression of TH (*p* < .0001, *p* < .001), and DA5‐CH had a more obvious effect (*p* < .0001). See Figure 6(b). Compared with the MPTP group, and compared with the MPTP group, both drugs reduced the production of α‐syn (*p* < .0001), and DA5‐CH was more effective (*p* < .0001). See Figure 6(c).

#### Immunohistochemical analysis

**FIGURE 7:**
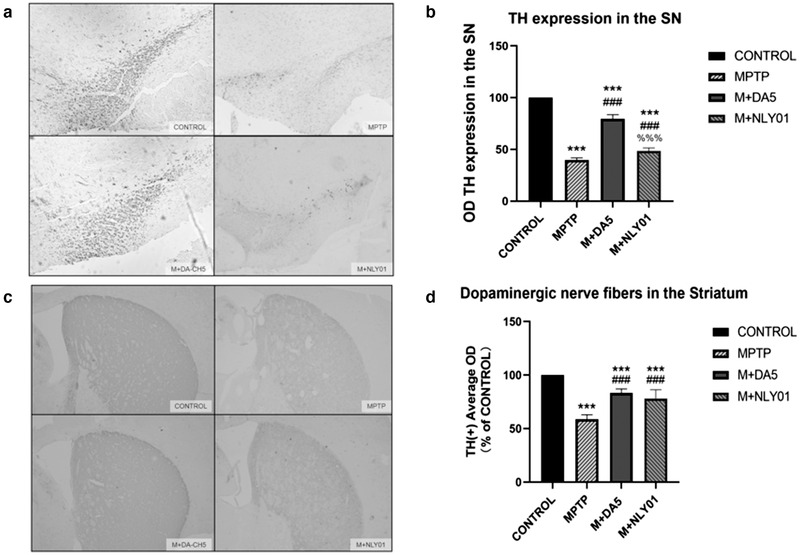
Immunohistochemical detection of the expression of TH in dopaminergic neurons (a) Staining of TH in the substantia nigra pars compacta. (b) Compared with the control group, the total number of TH‐positive cells in the MPTP group mice was significantly reduced to 39.65% (*p* < .0001); compared with the MPTP group, the total number of TH‐positive cells in the DA5‐CH group recovered to 79.61% (*p* < .0001), the total number of TH‐positive cells in the NLY01 group recovered slightly to 48.62%, and the DA5‐CH spray group recovered more significantly than the NLY01 group (*p* < .0001). (c) Staining of TH in the striatum. (d) Compared with the control group, the average optical density of TH staining in the MPTP group decreased to 58.73% (*p* < .0001); compared with the MPTP group, the average optical density of TH staining in the DA5‐CH group and the NLY01 group. The density recovered to 83.36% and 78.07% respectively (*p* < .0001), and the two groups were not statistically different compared with the control group (*p* > .05); *** = *p* < .0001 compared with the control group; ### = *p* < .0001 compared with the MPTP group; %%% = *p* < .0001 compared with the M+DA5‐CH group; Shown are representative images. Scale bar is 200 μm. *n* = 6 per group

In the substantia nigra of the midbrain, one‐way ANOVA found that: compared with the control group, the total number of TH‐positive cells in the MPTP group was significantly reduced to 39.65% (*p*<.0001); compared with the MPTP group, the DA5‐CH group was smaller. The total number of TH‐positive cells in the mice recovered to 79.61% (*p* < .0001), the total number of TH‐positive cells in the NLY01 group recovered to 48.62%, and the DA5‐CH group recovered more significantly than the NLY01 group (*p* < .0001). See Figure 7.

In the midbrain striatum, a one‐way ANOVA found that: compared with the control group, the average optical density of TH staining in the MPTP group decreased to 58.73% (*p*<.0001); compared with the MPTP group, DA5‐CH the average optical density of TH staining of mice in the group and NLY01 group recovered to 83.36% and 78.07%, respectively (*p*<.0001). There was no statistical significance between the two drug groups and the control group (*p*>.05). See Figure 7.

### 3.5 TLR4/NF‐κB signaling and expression levels of NF‐κB and TNF‐α

**FIGURE 8:**
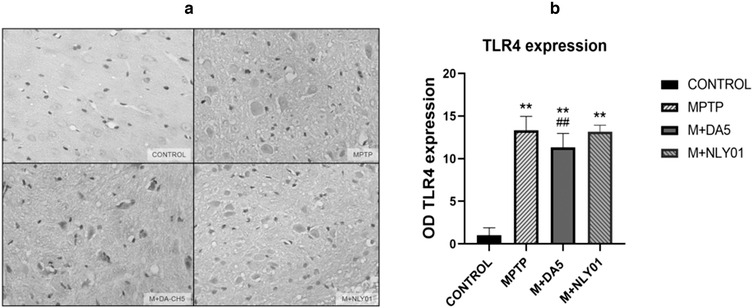
Compared with the control group, the number of TLR4 positive cells in the MPTP group increased significantly (*p* < .0001). Compared with the MPTP group, the decrease in the number of TLR4 positive cells in the DA5‐CH intervention group was statistically significant (*p* < .05), the number of TLR4 positive cells in the NLY01 group did not change (*p* > .05). ** = *p* < .01 compared with the control group; ## = *p* < .01 compared with the MPTP group. Shown are representative images, Scale bar is 50 μm. *n* = 6 per group

**FIGURE 9:**
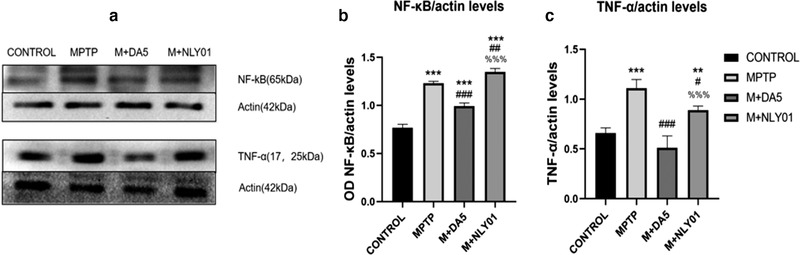
(a) Expression of NF‐κB and TNF‐α in brain tissue, sample western blots. (b) Compared with the control group, the expression level of NF‐κB in the MPTP group mice was significantly increased (*p* < .0001); compared with the MPTP group, the expression level of NF‐κB in the DA5‐CH intervention group and the NLY01 intervention group was increased (*p* < .0001, *p* < .01). The DA5‐CH group showed reduced NF‐κB expression level but the NLY01 intervention group did not (*p* < .0001). (c) Compared with the control group, the TNF‐α expression level in the MPTP group was significantly increased (*p* < .0001); compared with the MPTP group, the TNF‐α expression level of the mice in the DA5‐CH intervention group was significantly decreased (*p* < .0001), the increase in the expression level of NF‐κB in mice in the NLY01 intervention group was significant (*p* < .05), and the decrease in the expression level of TNF‐α in the DA5‐CH intervention group was more significant than that in the NLY01 intervention group (*p* < . 0001). *** = *p* < .0001 compared with the control group; ** = *p* < .01 compared with the control group; ### = *p* < .0001 compared with the MPTP group; ## = *p* < .01 compared with the MPTP group; # = *p* < .05 compared with the MPTP group; %%% = *p* < .0001 compared with the M+DA5‐CH group. *n* = 3 per group

In order to confirm whether DA5‐CH targeting inhibition of microglia activation is mediated by the TLR4/NF‐κB signaling pathway, we analyzed TLR4, NF‐κB and TNF in MPTP‐induced PD mice by Western blot and immunohistochemical‐expression level of TLR4. As shown in Figures 8 and 9, the expression levels of TLR4, NF‐κB and TNF‐α were significantly upregulated after MPTP injection. DA5‐CH down‐regulated the expression of TLR4, NF‐κB and TNF‐α, while NLY01 only reduced the expression level of NF‐kB and TNF‐α.

#### 3.5.1 DA5‐CH can reduce the expression of TLR4 in the midbrain

In the striatum, one‐way ANOVA found that: compared with the MPTP group, the number of TLR4 positive cells in the DA5‐CH intervention group decreased statistically (p<.05), and the TLR4 positive cells in the NLY01 intervention group the change in cell number was not statistically significant (p > .05). See Figure 8.

#### 3.5.2 NF‐κB and downstream inflammatory factor TNF‐α levels

##### Western blot assay

In the midbrain tissue, single‐factor ANOVA found that compared with the MPTP group, the NF‐κB expression level of the DA5‐CH intervention group and the NLY01 intervention group increased significantly (*p*<.0001, *p*<.01). The DA5‐CH intervention group had a more significant NF‐κB expression level than the NLY01 intervention group (*p*<.0001). See Figure 9(b). Compared with the MPTP group, the TNF‐α expression level of the DA5‐CH intervention group mice was significantly decreased (*p* < .0001), and the NF‐κB expression level of the NLY01 intervention group mice increased statistically (*p* < .05), the DA5‐CH spraying group had a more significant decrease in TNF‐α expression than the NLY01 intervention group (*p*<.0001). See Figure 9(c).

**FIGURE 10:**
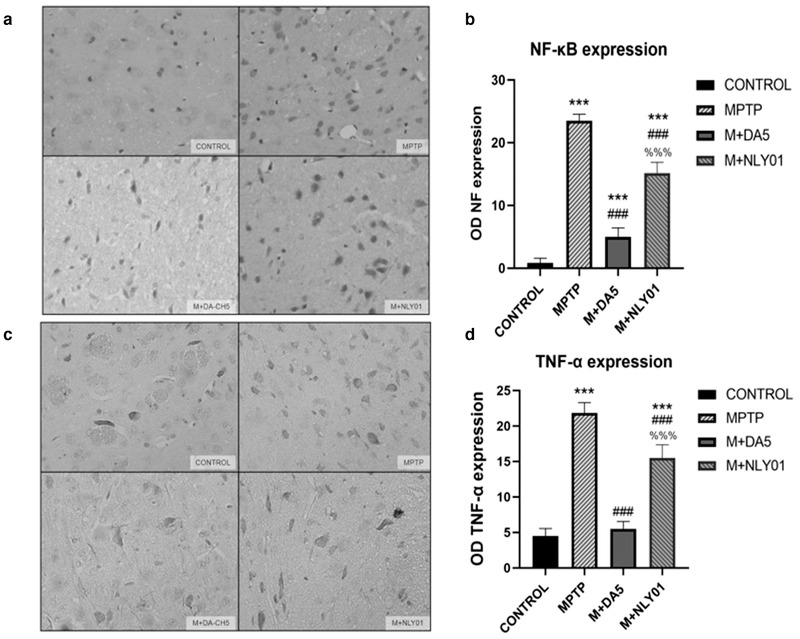
(a) Sample micrographs of NF‐κB cells in the striatum. (b) Compared with the control group, the number of NF‐κB positive cells in the MPTP group mice was significantly increased (*p* < .0001); compared with the MPTP group, the NF‐κB positive cells in the DA5‐CH intervention group and the NLY01 intervention group. The number of NF‐κB positive cells in the DA5‐CH intervention group was significantly reduced (*p* < .0001) compared with the NLY01 intervention group (*p* < .0001).(c) Sample micrographs of TNF‐α cells in the striatum. (d) Compared with the control group, the number of TNF‐α positive cells in the MPTP group was significantly increased (*p* < .0001); compared with the MPTP group, the TNF‐α positive cells in the DA5‐CH intervention group and the NLY01 intervention group was reduced. The number of TNF‐α‐positive cells decreased significantly (*p* < .0001) in the DA5‐CH intervention group than in the NLY01 intervention group (*p* < .0001). *** = *p* < .0001 compared with the control group; ### = *p* < .0001 compared with the MPTP group; %%% = *p* < .0001 compared with the M+DA5‐CH group. Shown are representative images, Scale bar is 50 μm, *n* = 6 per group

*Immunohistochemical analysis*. In the substantia nigra of the midbrain, one‐way ANOVA found that: compared with the MPTP group, the number of NF‐κo positive cells in the DA5‐CH intervention group and the NLY01 drug group was significantly reduced (*p* < .0001), DA5‐CH intervention when *c*ompared with the NLY01 intervention group, the number of NF‐κi positive cells in the mice in the NLY01 intervention group decreased more significantly (*p* < .0001). See Figure 10(b). Compared with the MPTP group, the number of TNF‐α positive cells in the DA5‐CH intervention group and the NLY01 intervention group was significantly reduced (*p*<.0001), and the number of TNF‐d the NLY01 ints in the DA5‐CH group were fewer than that of the NLY01 group (*p* < .0001). See Figure 10(d).

### 3.6 Activation of microglia and astrocytes, levels of pro‐inflammatory cytokines and anti‐inflammatory factors

The expression of microglia marker Iba‐1, microglia related anti‐inflammatory factor TGF‐β1 and astrocyte marker GFAP were detected by immunohistochemical staining method and Western blot method. One‐way ANOVA found that there were differences between all the groups, and the LSD comparison test between the groups found that the differences between the groups were significant. The results showed that compared with the control group, MPTP had a significant activation effect on microglia and astrocytes, and significantly reduced the expression of TGF‐β1.

#### Western blot assay

**FIGURE 11:**
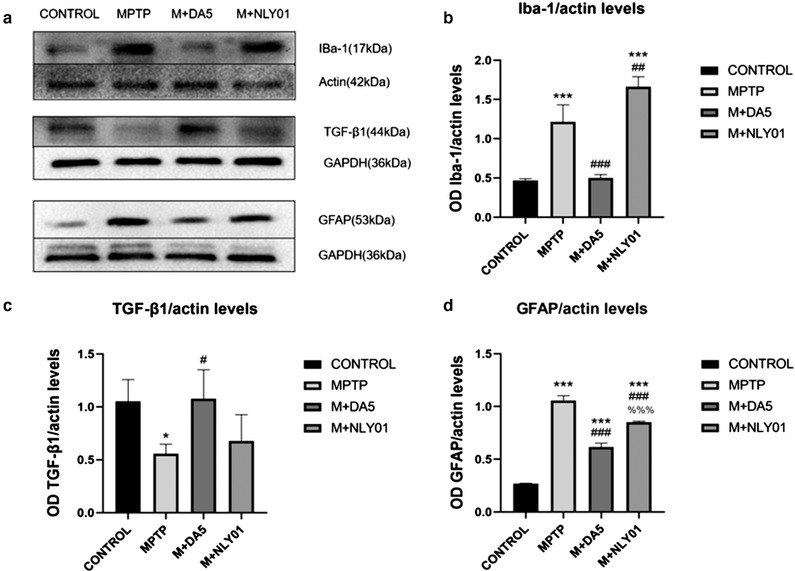
(a) Iba‐1, TGF‐β1 and GFAP western blot samples. (b) In the midbrain tissue, a one‐way analysis of variance found that: compared with the control group, the Iba‐1 expression level of the MPTP group mice was significantly Increased (*p*<.0001); compared with the MPTP group, the DA5‐CH intervention group. The expression level of Iba‐1 decreased significantly (*p* < .0001), while the expression level of Iba‐1 in the NLY01 intervention group mice was significantly Increased (*p* < .01). (c) In the midbrain tissue, one‐way analysis of variance found that: compared with the control group, the TGF‐β1 expression level of the MPTP group decreased (*p* < .05); compared with the MPTP group, DA5‐CH. The expression level of TGF‐β1 in the intervention group was statistically significant (*p* < .0001). (d) In the midbrain tissue, one‐way analysis of variance found that: compared with the control group, the GFAP expression level of the MPTP group mice was significantly Increased (*p*<.0001); Compared with the MPTP group,the GFAP expression level of mice in the the DA5‐CH intervention group and NLY01 intervention group was increased significantly (*p* < .0001), and the expression level of DA5‐CH intervention group was more significant than that of NLY01 intervention group (*p* < .0001). *** = *p* < .0001 compared with the control group; * = *p* < .05 compared with the control group; ### = *p* < .0001 compared with the MPTP group; ## = *p* < .01 compared with the MPTP group; # = *p* < .05 compared with the MPTP group; %%% = *p* < .0001 compared with the M+DA5‐CH group. *n* = 3 per group

In midbrain tissues, single‐factor ANOVA found that: compared with the MPTP group, the Iba‐1 expression level in the DA5‐CH group was significantly decreased (*p*<.0001), while the Iba‐1 expression level in the NLY01 drug group mice is significantly increased (*p* < .01). See Figure 11(b). Compared with the MPTP group, the expression level of TGF‐β1 in the DA5‐CH intervention group was statistically significant (*p* < .0001). See Figure 11(c). Compared with the MPTP group, the GFAP expression level of the mice in the DA5‐CH intervention group and the NLY01 intervention group was significantly decreased (*p* < .0001), and the expression level of the DA5‐CH group was more significantly lower than that of the NLY01 intervention group (*p* < .0001). See Figure 11(d).

#### Immunohistochemical analysis

**FIGURE 12:**
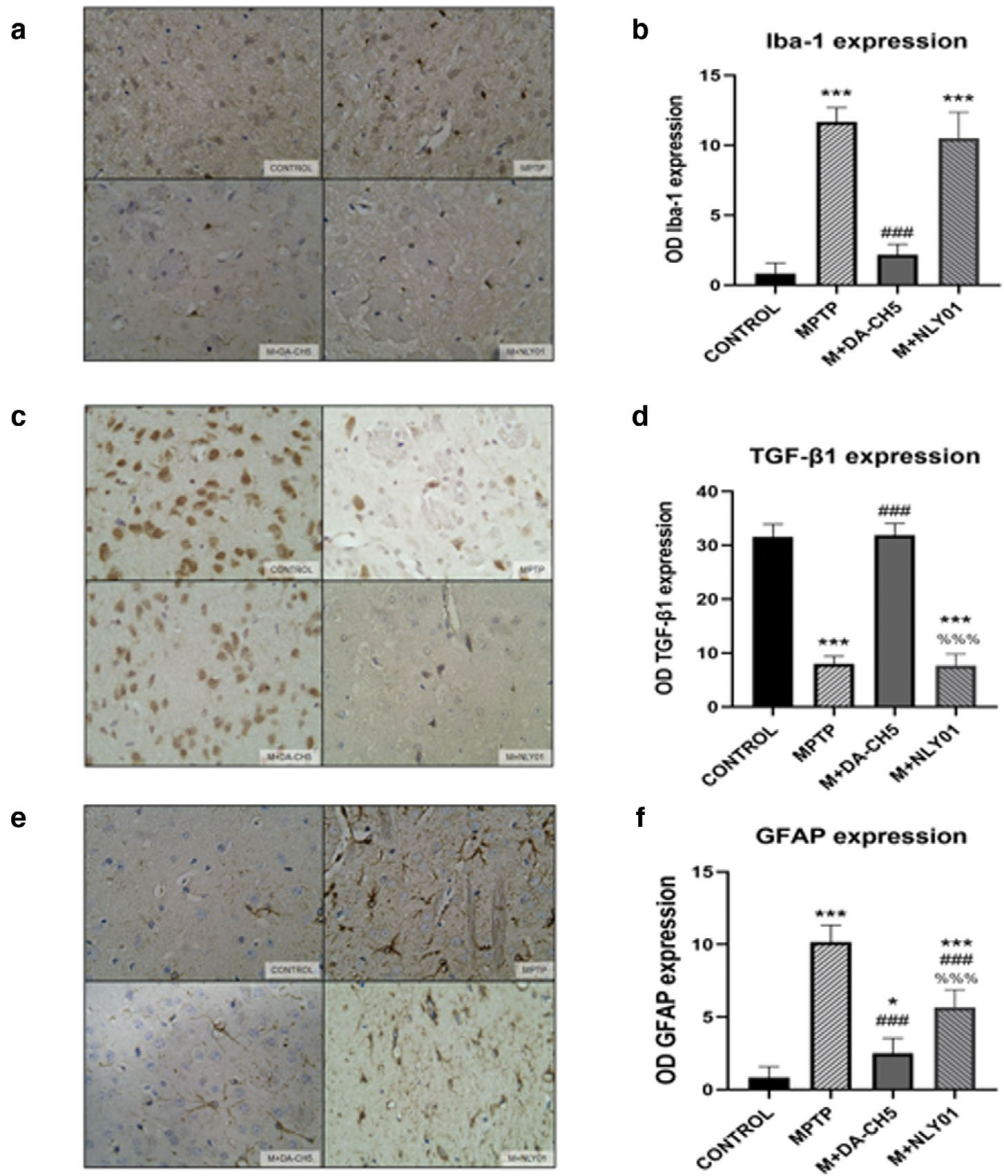
(a) Staining of activated microglia (Iba‐1) in the substantia nigra pars compact area. (b) Compared with the control group, the number of Iba‐1 positive cells in the MPTP group was significantly increased (*p* < .0001); compared with the MPTP group, the number of Iba‐1 positive cells in the DA5‐CH intervention group was significantly reduced (*p* < 0 0001), there was no statistical difference in the number of Iba‐1 positive cells in the NLY01 intervention group (*p* > .05).(c) Staining phase of TGF‐β1 cells. (d) Compared with the control group, the number of TGF‐β1 positive cells in the MPTP group was significantly reduced (*p* < .0001); compared with the MPTP group, the number of TGF‐β1 positive cells in the DA5‐CH intervention group was significantly increased (*p* < .0001). (e) Staining of GFAP cells. (f) Compared with the control group, the number of GFAP‐positive cells in the MPTP group increased significantly (*p* < .0001); compared with the MPTP group, the number of GFAP‐positive cells in the DA5‐CH intervention group and the NLY01 intervention group decreased significantly (*p* < .0001), the DA5‐CH intervention group reduced the number of GFAP positive cells more significantly than the NLY01 intervention group (*p* < .0001). *** = *p* < .0001 compared with the control group; ** = *p* < .01 compared with the control group; * = *p* < .05 compared with the control group; ### = *p* < .0001 compared with the MPTP group; ## = *p* < .01 compared with the MPTP group; %%% = *p* < .0001 compared with the M+DA5‐CH group. Shown are representative images, scale bar is 50 μm. *n* = 6 per group

In the midbrain tissue, single‐factor ANOVA found that: compared with the MPTP group, the number of Iba‐1 positive cells in the DA5‐CH intervention group was significantly reduced (*p*<.0001), and the number of Iba‐1 positive cells in the NLY01 intervention group changed. There is no statistical difference (*p* > .05). See Figure 12(b). Compared with the MPTP group, the number of TGF‐β1 positive cells in the DA5‐CH intervention group increased significantly (*p*<.0001). See Figure 12(d). Compared with the MPTP group, the number of GFAP positive cells in the DA5‐CH intervention group and the NLY01 spraying group was significantly reduced (*p* < .0001), and the DA5‐CH intervention group decreased more significantly than the NLY01 intervention group GFAP positive cell number (*p* < .0001). See Figure 12(f).

### 3.7 Pro‐ inflammatory factors IL‐6 and IL‐1β and anti‐inflammatory factor IL‐10

**FIGURE 13:**
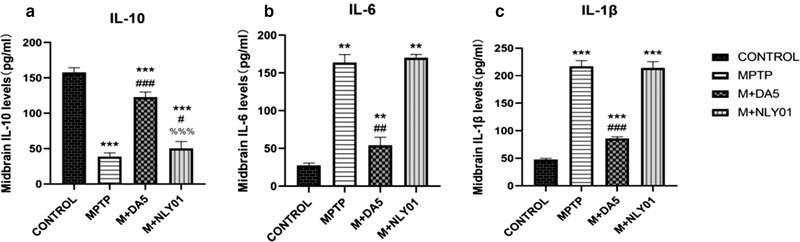
Analysis of the expression levels of IL‐10, IL‐6 and IL‐1β cytokines in the different groups. (a) Compared with the control group, the IL‐10 expression level of mice in the MPTP group was significantly reduced (*p* < .0001); compared with the MPTP group, the IL‐10 expression level of the mice in the DA5‐CH intervention group was significantly increased (*p* < .0001), the IL‐10 expression level of mice in the NLY01 intervention group decreased slightly (*p* < .05), and the IL‐10 expression level of mice in the DA5‐CH intervention group decreased more (*p* < .0001). (b) Compared with the control group, the IL‐6 expression level of mice in the MPTP group was significantly increased (*p*<.0001); compared with the MPTP group, the IL‐6 expression level of the DA5‐CH intervention group was significantly reduced (*p*<.0001), while the IL‐6 expression level of mice in the NLY01 intervention group was not statistically significant (*p* > .05). (c) Compared with the control group, the IL‐1β expression level of mice in the MPTP group was significantly increased (*p* < .0001); compared with the MPTP group, the IL‐1β expression level of the mice in the DA5‐CH intervention group was significantly reduced (*p* < .0001), the IL‐1β expression level changes of mice in the NLY01 intervention group were not statistically significant (*p* > .05). *** = *p* < .0001 compared with the control group; ** = *p* < .01 compared with the control group; ### = *p* < .0001 compared with the MPTP group; ## = *p* < .01 compared with the MPTP group; # = *p* < .05 compared with the MPTP group; %%% = *p* < .0001 compared with the M+DA5‐CH group. *n* = 6 per group

In the midbrain tissue, single‐factor ANOVA found that: compared with the MPTP group, the IL‐10 expression level of the DA5‐CH intervention group was significantly increased (*p*<.0001), and the IL‐10 expression of the NLY01 intervention group was slightly increased. The level reduction was statistically significant (*p* < .05), and the IL‐6 expression level of mice in the DA5‐CH intervention group decreased more significantly (*p* < .0001). Compared with the MPTP group, the IL‐1β expression level of mice in the DA5‐CH intervention group was significantly reduced (*p* < .0001), and the IL‐1β expression level of the mice in the NLY01 intervention group was not statistically significant (*p* > .05). See Figure 13.

### 3.8 Levels of glial cell‐derived neurotrophic factor (GDNF) and brain‐derived neurotrophic factor (BDNF)

#### Immunohistochemical analysis

**FIGURE 14:**
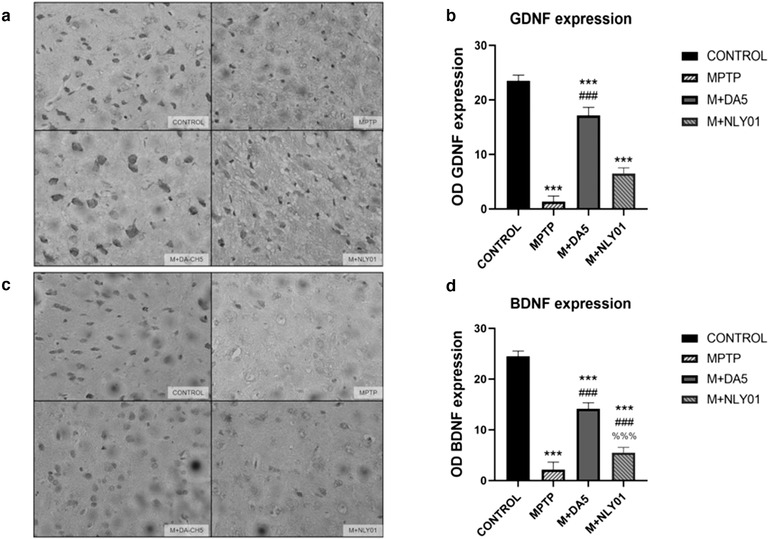
(a) Staining of GDNF in the striatum. (b) Compared with the control group, the number of GDNF positive cells in the MPTP group was significantly reduced (*p* < .0001); compared with the MPTP group, the number of GDNF positive cells in the DA5‐CH intervention group was significantly reduced (*p* < .0001), There was no statistical difference in the number of GDNF‐positive cells in the NLY01 intervention group (*p* > .05). (c) Stained image of BDNF cells in the striatum. (d) Compared with the control group, the number of BDNF positive cells in the MPTP group was significantly reduced (*p* < .0001); compared with the MPTP group, the number of BDNF positive cells in the DA5‐CH intervention group and the NLY01 intervention group was significantly reduced (*p* < .0001), the DA5‐CH intervention group had a more significant decrease in the number of BDNF positive cells than the NLY01 intervention group (*p* < .0001). *** = *p* < .0001 compared with the control group; ** = *p* < .01 compared with the control group; * = *p* < .05 compared with the control group; ### = *p*<.0001 compared with the MPTP group; %%% = *p* < .0001 compared with the M+DA5‐CH group. Shown are representative images, scale bar is 50 μm. *n* = 6 per group

In midbrain tissues, one‐way ANOVA found that, compared with the MPTP group, the number of GDNF positive cells in the DA5‐CH intervention group was significantly reduced (*p* < .0001), and the number of GDNF positive cells in the NLY01 intervention group was not statistically changed. Difference (*p* > .05). See Figure 14(b). Compared with the MPTP group, the number of BDNF positive cells in the DA5‐CH intervention group and the NLY01 intervention group was significantly reduced (*p* < .0001), and the number of BDNF positive cells in the DA5‐CH intervention group was more significantly reduced than that of the NLY01 intervention group (*p* < .0001). See Figure 14(d).

## 4 DISCUSSION

PD is a degenerative disease of the nervous system with complex mechanisms. It has now caused a high burden on the society, and its etiology and pathogenesis are still unclear. The MPTP mouse model is a classic animal model of PD which recapitulates the main pathological features of PD (Bove & Perier, 2012). MPTP is a fat‐soluble agent that can quickly pass through the blood‐brain barrier. It is taken up via the dopamine transporter and kills dopaminergic neurons through oxidative damage and inhibition of mitochondrial respiratory chain complexes, resulting in a decrease in dopaminergic neurons, and motor and non‐motor disorders that resemble PD (Bove & Perier, 2012; Gerlach et al., 1991). Among them, the reduction of striatal dopamine level is an important factor leading to characteristic motor symptoms, which are also the key to the diagnosis of PD. After intraperitoneal injection, MPTP is converted into the positively charged toxic metabolite 1‐methyl‐4‐phenylpyridine (MPP+) by monoamine oxidase‐B (MAO‐B). MPP+ causes a range of effects, which may involve mechanisms such as chronic inflammation, reduced energy utilization, apoptosis, and mitochondrial oxidation (Kopin & Markey, 1988; Nakamura & Vincent, 1986).

GLP‐1 and GIP are peptide hormones with neuroprotective properties. A range of receptor agonists including dual GLP‐1/GIP receptor agonists have shown clear neuroprotective effects in animal models of PD. They act as growth factors that normalize energy utilization, cell repair, synaptic activity and reduce the chronic inflammation response (Hölscher, 2018). Importantly, a GLP‐1 receptor agonist has shown good protective and disease‐modifying effectsin PD patients as shown in reduced disease progression even 3 months after drug administration had stopped (Athauda et al., 2017), and improvement of growth‐factor signaling in the brain (Athauda et al., 2019). Compared with a single GLP‐1 or GIP receptor agonist, the new dual GLP‐1/GIP receptor agonist DDA5‐CH with a TAT sequence attached, which crosses the blood‐brain barrier at an enhanced rate compared to single GLP‐1 receptor agonists (Li et al., 2020; Zhang et al., 2020), exhibits superior neuroprotection in different animal models (Feng et al., 2018; Zhang et al., 2020). The results show that crossing the BBB is an important property for successful drug action. NLY01 is an exendin‐4 peptide with a 40kDA pegylation (Yun et al., 2018). We and others have shown that pegylated peptide hormones do not readily cross the BBB (Salameh et al., 2020; Zhang et al., 2020). We show here that NLY01 enters the brain only at very limited quantities, confirming earlier studies. NLY01 had been tested in mouse models of PD or inflammation, and using the same technique that we used here, they showed that NLY01 does not readily cross the BBB in wild‐type mice (Yun et al., 2018) (see Figure S2). This can explain the fact that NLY01 showed virtually no effects in the different measurements presented here, while non‐pegylated exendin‐4 does show good neuroprotective effects in PD (Athauda & Foltynie, 2018; Bertilsson et al., 2008; Hölscher, 2016; Kim et al., 2009; Li et al., 2009). Non‐pegylated exendin‐4 enters the brain quickly and therefore can act in the CNS while NLY01 cannot (Salameh et al., 2020).

There is increasing evidence that impaired brain insulin signaling play a role in the pathogenesis of Parkinson's disease (Athauda & Foltynie, 2016; Bosco et al., 2012; Cheong et al., 2020). GLP‐1 analogues can improve insulin desensitization in the brain, which may be one of the mechanisms of action of these drugs (Gao et al., 2013; Li et al., 2020; Shi et al., 2017). In addition, MPTP treatment can induce chronic inflammation of the brain and cause microglia to produce a large number of inflammatory cytokines (Bove & Perier, 2012). Chronic inflammation of the CNS is a key driver of PD and can be facilitated by diabetes (Cheong et al., 2020; Clark & Vissel, 2014; Ferrari & Tarelli, 2011; Hirsch & Hunot, 2009). In the early stage of PD, microglia are not activated into the chronic inflammatory state and help to protect neurons and clear up debris. As the disease progresses, the release of inflammatory factors gradually increases, leading to the activation of microglia (Blandini, 2013). Among them, the chronic inflammation process can lead to a decrease in the level of microglia‐derived GLP‐1. Then, the inflammatory factors activate astrocytes, and like microglia, astrocytes become reactive astrocytes after being stimulated, which can produce pro‐inflammatory cytokines in vivo and in vitro (Ferrari & Tarelli, 2011; Orr et al., 2002). Reactive astrogliosis is characterized by increased expression of glial fibrillary acidic protein (GFAP) and enlarged cell bodies in many inflammatory pathways. Previous studies have elucidated the significance of NF‐κB in the central nervous system. NF‐κB can be regulated by TLRs. Among them, the activation of microglia through TLR4/NF‐κB plays an important role in initiating the innate immune response (Ferrari & Tarelli, 2011). The two glial cell‐specific inflammatory factors are TNF‐α, IL‐6 and IL‐1β, which can directly activate cytokines to induce dopaminergic neuron apoptosis. It has been reported that in the MPTP model of PD, the inflammatory response has been determined and the pro‐inflammatory cytokine TNF‐α level has been detected to increase (Bove & Perier, 2012; Schintu et al., 2009; Yuan et al., 2017; Zhang et al., 2019). The results of our study demonstrate that DA5‐CH can reduce the chronic inflammation response while NLY01 is not effective at the dose tested. In spite of a recent publication that claims good anti‐inflammatory effects of NLY01 (Yun et al., 2018), our study clearly demonstrates that the effect is very limited.

When comparing the two drugs in motor tasks and muscle strength assessments, DA5‐CH showed robust improvements, while NLY01 was weak in improving PD related motor impairments. Nowadays, in clinical practice, gait is an important indicator for evaluating PD dyskinesia, which can reflect the stability and coordination of walking. DA5‐CH significantly improved the motor behavior disorder of MPTP mice, but NLY01 was not effective. When analyzing the effects of the drugs on dopamine synthesis in the SN, specifically Tyrosine hydroxylase (TH) which is the rate‐limiting enzyme in the dopamine synthesis pathway that is responsible for converting tyrosine to L‐dopa, we showed that DA5‐CH was effective in normalizing the levels of TH expression after MPTP treatment, while NLY01 treatment was ineffective in increasing the level of TH‐positive cells in the substantia nigra of MPTP treated mice.

GDNF is a key growth factor for neuronal development and repair and has been shown to have a specific effect on midbrain dopaminergic neurons that show the earliest degeneration in PD. Infusion of GDNF into the striatum into the ventricle and brain parenchyma can improve neurodegeneration. The striatum and substantia nigra dopamine levels were significantly increased (Lin et al., 1993). In addition, brain‐derived neurotrophic factor (BDNF) is widely expressed throughout the brain and peripheral nervous system (He et al., 2013), and the strongest association with it is motor‐related neurons, including the primary motor cortex, basal ganglia and substantia nigra. BDNF can improve the survival of dopaminergic neurons, and the lack of BDNF can lead to the occurrence of PD (Frazzitta et al., 2014; Zuccato & Cattaneo, 2009). Studies have shown that the reduced level of BDNF is related to the severity of the disease, suggesting that BDNF may play a role in the pathogenesis of the disease (Allen et al., 2013; Frazzitta et al., 2014; He et al., 2013). This study proves that DA5‐CH can increase the expression of GDNF and BDNF in midbrain tissue, and provide a new direction for the study of the therapeutic mechanism of new GLP‐1/GIP dual agonists for Parkinson's disease. In contrast, NLY01 was not effective.

It is believed that abnormal accumulations of α‐Syn in the brain plays a role in the development of PD. We have previously shown that GLP‐1 and GLP‐1/GIP receptor agonists can normalize autophagy in neurons and can lower the levels of α‐Syn (Panagaki et al., 2017, 2018; Zhang et al., 2019). NLY01 did not reduce levels of α‐Syn at the dose chosen. In contrast, DA5‐CH lowered levels significantly. We believe the reason is linked to the inability of NLY01 to effectively cross the BBB. NLY01 is currently being tested in a phase II clinical trial in PD patients (NCT04154072), and it will be possible to compare the outcome with the effects of un‐pegylated exendin‐4 from a study that has been finished and that showed good effects (Athauda et al., 2017, 2019).

In conclusion, we were able to demonstrate that the novel dual GLP‐1/GIP receptor agonist DA5‐CH which has a CPT modification to cross the BBB at an enhanced rate is capable of improving a range of key pathological features of PD in the MPTP mouse model. The pegylated peptide NLY01 did not show comparable effects and would need to be dose at much higher doses, enhancing side effects in the periphery. DA5‐CH appears to be a potent drug with great potential as a novel treatment for PD.

## CONFLICT OF INTEREST

CH is a named inventor on several patents that cover the use of dual GLP‐1/GIP receptor agonists as a treatment for PD. He is furthermore the CSO of the biotech company Kariya Pharmaceuticals. The other authors do not declare a conflict of interest.

### PEER REVIEW

The peer review history for this article is available at https://publons.com/publon/10.1002/brb3.2231.

## References

[brb32231-bib-0001] Allen, S. J., Watson, J. J., Shoemark, D. K., Barua, N. U., & Patel, N. K. (2013). GDNF, NGF and BDNF as therapeutic options for neurodegeneration. Pharmacology & Therapeutics, 138(2), 155‐175. 10.1016/j.pharmthera.2013.01.004 23348013

[brb32231-bib-0002] Athauda, D., & Foltynie, T. (2016). Insulin resistance and Parkinson's disease: A new target for disease modification? Progress in Neurobiology, 145, 98‐120. 10.1016/j.pneurobio.2016.10.001 27713036

[brb32231-bib-0003] Athauda, D., & Foltynie, T. (2018). Protective effects of the GLP‐1 mimetic exendin‐4 in Parkinson's disease. Neuropharmacology, 136(Pt B), 260‐270. 10.1016/j.neuropharm.2017.09.023 28927992

[brb32231-bib-0004] Athauda, D., Gulyani, S., Karnati, H., Li, Y., Tweedie, D., Mustapic, M., Chawla, S., Chowdhury, K., Skene, S. S., Greig, N. H., Kapogiannis, D., & Foltynie, T. (2019). Utility of neuronal‐derived exosomes to examine molecular mechanisms that affect motor function in patients with parkinson disease: A secondary analysis of the exenatide‐PD trial. JAMA Neurology, 76, 420‐429. 10.1001/jamaneurol.2018.4304 30640362 PMC6459135

[brb32231-bib-0005] Athauda, D., Maclagan, K., Skene, S. S., Bajwa‐Joseph, M., Letchford, D., Chowdhury, K., Hibbert, S., Budnik, N., Zampedri, L., Dickson, J., Li, Y., Aviles‐Olmos, I., Warner, T. T., Limousin, P., Lees, A. J., Greig, N. H., Tebbs, S., & Foltynie, T. (2017). Exenatide once weekly versus placebo in Parkinson's disease: A randomised, double‐blind, placebo‐controlled trial. The Lancet, 390(17), 1664‐1675. 10.1016/S0140-6736(17)31585-4 PMC5831666 28781108

[brb32231-bib-0006] Aviles‐Olmos, I., Limousin, P., Lees, A., & Foltynie, T. (2013). Parkinson's disease, insulin resistance and novel agents of neuroprotection. Brain, 136(Pt 2), 374‐384. 10.1093/brain/aws009 22344583

[brb32231-bib-0007] Bengoa‐Vergniory, N., Roberts, R. F., Wade‐Martins, R., & Alegre‐Abarrategui, J. (2017). Alpha‐synuclein oligomers: A new hope. Acta Neuropathologica, 134(6), 819‐838. 10.1007/s00401-017-1755-1 28803412 PMC5663814

[brb32231-bib-0008] Bertilsson, G., Patrone, C., Zachrisson, O., Andersson, A., Dannaeus, K., Heidrich, J., Kortesmaa, J., Mercer, A., Nielsen, E., Rönnholm, H., & Wikstrom, L. (2008). Peptide hormone exendin‐4 stimulates subventricular zone neurogenesis in the adult rodent brain and induces recovery in an animal model of Parkinson's disease. Journal of Neuroscience Research, 86(2), 326‐338. 10.1002/jnr.21483 17803225

[brb32231-bib-0009] Blandini, F. (2013). Neural and immune mechanisms in the pathogenesis of Parkinson's disease. Journal of Neuroimmune Pharmacology: The Official Journal of the Society on NeuroImmune Pharmacology, 8(1), 189‐201. 10.1007/s11481-013-9435-y 23378275

[brb32231-bib-0010] Bosco, D., Plastino, M., Cristiano, D., Colica, C., Ermio, C., De Bartolo, M., Mungari, P., Fonte, G., Consoli, D., Consoli, A., & Fava, A. (2012). Dementia is associated with insulin resistance in patients with Parkinson's disease. Journal of the Neurological Sciences, 315(1‐2), 39‐43. 10.1016/j.jns.2011.12.008 22265943

[brb32231-bib-0011] Bove, J., & Perier, C. (2012). Neurotoxin‐based models of Parkinson's disease. Neuroscience, 211, 51‐76. 10.1016/j.neuroscience.2011.10.057 22108613

[brb32231-bib-0012] Caggiu, E., Arru, G., Hosseini, S., Niegowska, M., Sechi, G., Zarbo, I. R., & Sechi, L. A. (2019). Inflammation, infectious triggers, and parkinson's disease. Frontiers in Neurology, 10, 122. 10.3389/fneur.2019.00122 30837941 PMC6389614

[brb32231-bib-0013] Cheong, J. L. Y., de Pablo‐Fernandez, E., Foltynie, T., & Noyce, A. J. (2020). The association between type 2 diabetes mellitus and Parkinson's disease. Journal of Parkinson's Disease, 10(3), 775‐789. 10.3233/JPD-191900 PMC7458510 32333549

[brb32231-bib-0014] Clark, I. A., & Vissel, B. (2014). Inflammation‐sleep interface in brain disease: TNF, insulin, orexin. Journal of Neuroinflammation, 11, 51. 10.1186/1742-2094-11-51 24655719 PMC3994460

[brb32231-bib-0015] Darsalia, V., Mansouri, S., Ortsater, H., Olverling, A., Nozadze, N., Kappe, C., Iverfeldt, K., Tracy, L. M., Grankvist, N., Sjöholm, Å., & Patrone, C. (2012). Glucagon‐like peptide‐1 receptor activation reduces ischaemic brain damage following stroke in Type 2 diabetic rats. Clinical Science, 122(10), 473‐483. 10.1042/CS20110374 22150224 PMC3268352

[brb32231-bib-0016] Darsalia, V., Ortsäter, H., Olverling, A., Darlöf, E., Wolbert, P., Nyström, T., Klein, T., Sjöholm, Å., & Patrone, C. (2013). The DPP‐4 inhibitor linagliptin counteracts stroke in the normal and diabetic mouse brain: A comparison with glimepiride. Diabetes, 62(4), 1289‐1296. 10.2337/db12-0988 23209191 PMC3609599

[brb32231-bib-0017] During, M. J., Cao, L., Zuzga, D. S., Francis, J. S., Fitzsimons, H. L., Jiao, X., Bland, R. J., Klugmann, M., Banks, W. A., Drucker, D. J., & Haile, C. N. (2003). Glucagon‐like peptide‐1 receptor is involved in learning and neuroprotection. Nature Medicine, 9(9), 1173‐1179. 10.1038/nm919 12925848

[brb32231-bib-0018] Feng, P., Zhang, X., Li, D., Ji, C., Yuan, Z., Wang, R., Xue, G., Li, G., & Hölscher, C. (2018). Two novel dual GLP‐1/GIP receptor agonists are neuroprotective in the MPTP mouse model of Parkinson's disease. Neuropharmacology, 133, 385‐394. 10.1016/j.neuropharm.2018.02.012 29462693

[brb32231-bib-0019] Ferrari, C. C., & Tarelli, R. (2011). Parkinson's disease and systemic inflammation. Parkinson's Disease, 2011, 436813. 10.4061/2011/436813 PMC3049348 21403862

[brb32231-bib-0020] Finan, B., Ma, T., Ottaway, N., Muller, T. D., Habegger, K. M., Heppner, K. M., Kirchner, H., Holland, J., Hembree, J., Raver, C., Lockie, S. H., Smiley, D. L., Gelfanov, V., Yang, B., Hofmann, S., Bruemmer, D., Drucker, D. J., Pfluger, P. T., Perez‐Tilve, D., …, Tschop, M. H. (2013). Unimolecular dual incretins maximize metabolic benefits in rodents, monkeys, and humans. Science Translational Medicine, 5(209), 209ra151. 10.1126/scitranslmed.3007218 24174327

[brb32231-bib-0021] Foltynie, T., & Athauda, D. (2020). Diabetes, BMI, and Parkinson's. Movement Disorders, 35(2), 201‐203. 10.1002/mds.27941 32056307

[brb32231-bib-0022] Frazzitta, G., Maestri, R., Ghilardi, M. F., Riboldazzi, G., Perini, M., Bertotti, G., Boveri, N., Buttini, S., Lombino, F. L., Uccellini, D., Turla, M., Pezzoli, G., & Comi, C. (2014). Intensive rehabilitation increases BDNF serum levels in parkinsonian patients: A randomized study. Neurorehabilitation and Neural Repair, 28(2), 163‐168. 10.1177/1545968313508474 24213955

[brb32231-bib-0023] Gao, C., Liu, Y., Li, L., & Holscher, C. (2013). New animal models of Alzheimer's disease that display insulin desensitization in the brain. Reviews in the Neurosciences, 24(6), 607‐615. 10.1515/revneuro-2013-0034 24259244

[brb32231-bib-0024] Gerlach, M., Riederer, P., Przuntek, H., & Youdim, M. B. (1991). MPTP mechanisms of neurotoxicity and their implications for Parkinson's disease. European Journal of Pharmacology, 208(4), 273‐286. 10.1016/0922-4106(91)90073-Q 1815982

[brb32231-bib-0025] Graham, D. L., Durai, H. H., Trammell, T. S., Noble, B. L., Mortlock, D. P., Galli, A., & Stanwood, G. D. (2020). A novel mouse model of glucagon‐like peptide‐1 receptor expression: A look at the brain. Journal of Comparative Neurology, 528(14), 2445‐2470. 10.1002/cne.24905 PMC7392814 32170734

[brb32231-bib-0026] Hamilton, A., & Hölscher, C. (2009). Receptors for the insulin‐like peptide GLP‐1 are expressed on neurons in the CNS. Neuroreport, 20, 1161‐1166. 10.1097/WNR.0b013e32832fbf14 19617854

[brb32231-bib-0027] He, Y. Y., Zhang, X. Y., Yung, W. H., Zhu, J. N., & Wang, J. J. (2013). Role of BDNF in central motor structures and motor diseases. Molecular Neurobiology, 48(3), 783‐793. 10.1007/s12035-013-8466-y 23649659

[brb32231-bib-0028] Hirsch, E. C., & Hunot, S. (2009). Neuroinflammation in Parkinson's disease: A target for neuroprotection? Lancet Neurology, 8(4), 382‐397. 10.1016/S1474-4422(09)70062-6 19296921

[brb32231-bib-0029] Hölscher, C. (2016). GLP‐1 and GIP analogues as novel treatments for Alzheimer's and Parkinson's disease. Cardiovascular Endocrinology, 5(3), 93‐98. 10.1097/XCE.0000000000000087

[brb32231-bib-0030] Hölscher, C. (2018). Novel dual GLP‐1/GIP receptor agonists show neuroprotective effects in Alzheimer's and Parkinson's disease models. Neuropharmacology, 136, 251‐259. 10.1016/j.neuropharm.2018.01.040 29402504

[brb32231-bib-0031] Kastin, A. J., Akerstrom, V., & Pan, W. (2002). Interactions of glucagon‐like peptide‐1 (GLP‐1) with the blood‐brain barrier. Journal of Molecular Neuroscience, 18(1‐2), 7‐14. 10.1385/JMN:18:1-2:07 11931352

[brb32231-bib-0032] Kim, S., Moon, M., & Park, S. (2009). Exendin‐4 protects dopaminergic neurons by inhibition of microglial activation and matrix metalloproteinase‐3 expression in an animal model of Parkinson's disease. Journal of Endocrinology, 202(3), 431‐439. 10.1677/JOE-09-0132 19570816

[brb32231-bib-0033] Kim, T. H., Jiang, H. H., Lim, S. M., Youn, Y. S., Choi, K. Y., Lee, S., Chen, X., Byun, Y., & Lee, K. C. (2012). Site‐specific PEGylated Exendin‐4 modified with a high molecular weight trimeric PEG reduces steric hindrance and increases type 2 antidiabetic therapeutic effects. Bioconjugate Chemistry, 23(11), 2214‐2220. 10.1021/bc300265n 23116483

[brb32231-bib-0034] Kopin, I. J., & Markey, S. P. (1988). MPTP toxicity: Implications for research in Parkinson's disease. Annual Review of Neuroscience, 11, 81‐96. 10.1146/annurev.ne.11.030188.000501 3129982

[brb32231-bib-0035] Lee, C. H., Yan, B., Yoo, K. Y., Choi, J. H., Kwon, S. H., Her, S., Sohn, Y., Hwang, I. K., Cho, J. H., Kim, Y. ‐ M., & Won, M. H. (2011). Ischemia‐induced changes in glucagon‐like peptide‐1 receptor and neuroprotective effect of its agonist, exendin‐4, in experimental transient cerebral ischemia. Journal of Neuroscience Research, 89(7), 1103‐1113. 10.1002/jnr.22596 21472764 10.1002/jnr.22596

[brb32231-bib-0036] Li, C., Liu, W., Li, X., Zhang, Z., Qi, H., Liu, S., Yan, N., Xing, Y., Hölscher, C., & Wang, Z. (2020). The novel GLP‐1/GIP analogue DA5‐CH reduces tau phosphorylation and normalizes theta rhythm in the icv. STZ rat model of AD. Brain and Behavior, 10, e01505. 10.1002/brb3.1505 31960630 PMC7066337

[brb32231-bib-0037] Li, Y., Liu, W., Li, L., & Holscher, C. (2016). Neuroprotective effects of a GIP analogue in the MPTP Parkinson's disease mouse model. Neuropharmacology, 101, 255‐263. 10.1016/j.neuropharm.2015.10.002 26453962

[brb32231-bib-0038] Li, Y., Liu, W., Li, L., & Holscher, C. (2017). D‐Ala2‐GIP‐glu‐PAL is neuroprotective in a chronic Parkinson's disease mouse model and increases BNDF expression while reducing neuroinflammation and lipid peroxidation. European Journal of Pharmacology, 797, 162‐172. 10.1016/j.ejphar.2016.11.050 27913104

[brb32231-bib-0039] Li, Y., Perry, T., Kindy, M. S., Harvey, B. K., Tweedie, D., Holloway, H. W., Powers, K., Shen, H., Egan, J. M., Sambamurti, K., Brossi, A., Lahiri, D. K., Mattson, M. P., Hoffer, B. J., Wang, Y., & Greig, N. H. (2009). GLP‐1 receptor stimulation preserves primary cortical and dopaminergic neurons in cellular and rodent models of stroke and Parkinsonism. PNAS, 106, 1285‐1290. 10.1073/pnas.0806720106 19164583 PMC2633544

[brb32231-bib-0040] Lin, L. F., Doherty, D. H., Lile, J. D., Bektesh, S., & Collins, F. (1993). GDNF: A glial cell line‐derived neurotrophic factor for midbrain dopaminergic neurons. Science, 260(5111), 1130‐1132. 10.1126/science.8493557 8493557

[brb32231-bib-0041] Merchenthaler, I., Lane, M., & Shughrue, P. (1999). Distribution of pre‐pro‐glucagon and glucagon‐like peptide‐1 receptor messenger RNAs in the rat central nervous system. Journal of Comparative Neurology, 403(2), 261‐280. 10.1002/(SICI)1096-9861(19990111)403:2<261::AID-CNE8>3.0.CO;2-5 9886047

[brb32231-bib-0042] Miyake, Y., Tanaka, K., Fukushima, W., Sasaki, S., Kiyohara, C., Tsuboi, Y., Yamada, T., Oeda, T., Miki, T., Kawamura, N., Sakae, N., Fukuyama, H., Hirota, Y., Nagai, M., & Fukuoka Kinki Parkinson's Disease Study, G. (2010). Case‐control study of risk of Parkinson's disease in relation to hypertension, hypercholesterolemia, and diabetes in Japan. Journal of the Neurological Sciences, 293(1‐2), 82‐86. 10.1016/j.jns.2010.03.002 20347450

[brb32231-bib-0043] Nakamura, S., & Vincent, S. R. (1986). Histochemistry of MPTP oxidation in the rat brain: Sites of synthesis of the parkinsonism‐inducing toxin MPP+. Neuroscience Letters, 65(3), 321‐325. 10.1016/0304-3940(86)90282-X 3487052

[brb32231-bib-0044] Orr, C. F., Rowe, D. B., & Halliday, G. M. (2002). An inflammatory review of Parkinson's disease. Progress in Neurobiology, 68(5), 325‐340. 10.1016/S0301-0082(02)00127-2 12531233

[brb32231-bib-0045] Panagaki, T., Gengler, S., & Holscher, C. (2018). The novel DA3‐CH dual incretin restores endoplasmic reticulum stress and autophagy impairments to attenuate alzheimer‐like pathology and cognitive decrements in the APPSWE/PS1DeltaE9 mouse model. Journal of Alzheimers Disease, 66, 195‐218. 10.3233/JAD-180584 30282365

[brb32231-bib-0046] Panagaki, T., Michael, M., & Hölscher, C. (2017). Liraglutide restores chronic ER stress, autophagy impairments and apoptotic signalling in SH‐SY5Y cells. Scientific Reports, 7(1), 16158. 10.1038/s41598-017-16488-x 29170452 PMC5700973

[brb32231-bib-0047] Perry, V. H., Cunningham, C., & Holmes, C. (2007). Systemic infections and inflammation affect chronic neurodegeneration. Nature Reviews Immunology, 7(2), 161‐167. 10.1038/nri2015 17220915

[brb32231-bib-0048] Salameh, T. S., Rhea, E. M., Talbot, K., & Banks, W. A. (2020). Brain uptake pharmacokinetics of incretin receptor agonists showing promise as Alzheimer's and Parkinson's disease therapeutics. Biochemical Pharmacology, 180, 114187. 10.1016/j.bcp.2020.114187 32755557 PMC7606641

[brb32231-bib-0049] Scherrmann, J. M. (2002). Drug delivery to brain via the blood‐brain barrier. Vascular Pharmacology, 38(6), 349‐354. 10.1016/S1537-1891(02)00202-1 12529929

[brb32231-bib-0050] Schintu, N., Frau, L., Ibba, M., Caboni, P., Garau, A., Carboni, E., & Carta, A. R. (2009). PPAR‐gamma‐mediated neuroprotection in a chronic mouse model of Parkinson's disease. European Journal of Neuroscience, 29(5), 954‐963. 10.1111/j.1460-9568.2009.06657.x 19245367

[brb32231-bib-0051] Shi, L., Zhang, Z., Li, L., & Holscher, C. (2017). A novel dual GLP‐1/GIP receptor agonist alleviates cognitive decline by re‐sensitizing insulin signaling in the Alzheimer icv. STZ rat model. Behavioural Brain Research, 327, 65‐74. 10.1016/j.bbr.2017.03.032 28342971

[brb32231-bib-0052] Tansey, M. G., & Goldberg, M. S. (2010). Neuroinflammation in Parkinson's disease: Its role in neuronal death and implications for therapeutic intervention. Neurobiology of Disease, 37(3), 510‐518. 10.1016/j.nbd.2009.11.004 19913097 PMC2823829

[brb32231-bib-0053] Teramoto, S., Miyamoto, N., Yatomi, K., Tanaka, Y., Oishi, H., Arai, H., Hattori, N., & Urabe, T. (2011). Exendin‐4, a glucagon‐like peptide‐1 receptor agonist, provides neuroprotection in mice transient focal cerebral ischemia. Journal of Cerebral Blood Flow and Metabolism, 31(8), 1696‐1705. 10.1038/jcbfm.2011.51 21487412 PMC3170947

[brb32231-bib-0054] Togashi, H., Sasaki, M., Frohman, E., Taira, E., Ratan, R. R., Dawson, T. M., & Dawson, V. L. (1997). Neuronal (type I) nitric oxide synthase regulates nuclear factorkappaB activity and immunologic (type II) nitric oxide synthase expression. Proceedings of the National Academy of Sciences of the Usa, 94(6), 2676‐2680. 10.1073/pnas.94.6.2676 9122255 PMC20148

[brb32231-bib-0055] Xu, Q., Park, Y., Huang, X., Hollenbeck, A., Blair, A., Schatzkin, A., & Chen, H. (2011). Diabetes and risk of Parkinson's disease. Diabetes Care, 34(4), 910‐915. 10.2337/dc10-1922 21378214 PMC3064050

[brb32231-bib-0056] Yuan, Z., Li, D., Feng, P., Xue, G., Ji, C., Li, G., & Holscher, C. (2017). A novel GLP‐1/GIP dual agonist is more effective than liraglutide in reducing inflammation and enhancing GDNF release in the MPTP mouse model of Parkinson's disease. European Journal of Pharmacology, 812, 82‐90. 10.1016/j.ejphar.2017.06.029 28666800

[brb32231-bib-0057] Yun, S. P., Kam, T. I., Panicker, N., Kim, S., Oh, Y., Park, J. S., Kwon, S. ‐ H., Park, Y. J., Karuppagounder, S. S., Park, H., Kim, S., Oh, N., Kim, N. A., Lee, S., Brahmachari, S., Mao, X., Lee, J. H., Kumar, M., An, D., …, Ko, H. S. (2018). Block of A1 astrocyte conversion by microglia is neuroprotective in models of Parkinson's disease. Nature Medicine, 24(7), 931‐938. 10.1038/s41591-018-0051-5 PMC6039259 29892066

[brb32231-bib-0058] Zhang, L., Zhang, L., Li, L., & Holscher, C. (2019). Semaglutide is neuroprotective and reduces alpha‐synuclein levels in the chronic MPTP mouse model of parkinson's disease. Journal of Parkinson's Disease, 9(1), 157‐171. 10.3233/JPD-181503 30741689

[brb32231-bib-0059] Zhang, L., Zhang, L., Li, Y., Li, L., Melchiorsen, J., Rosenkilde, M., & Hölscher, C. (2020). The novel dual GLP‐1/GIP receptor agonist DA‐CH5 is superior to single GLP‐1 receptor agonists in the MPTP model of Parkinson's disease. Journal of Parkinson's Disease, 10, 523‐542. 10.3233/JPD-191768 31958096

[brb32231-bib-0060] Zhang, Z. Q., & Holscher, C. (2020). GIP has neuroprotective effects in Alzheimer and Parkinson's disease models. Peptides, 125, 170184. 10.1016/j.peptides.2019.170184 31705913

[brb32231-bib-0061] Zuccato, C., & Cattaneo, E. (2009). Brain‐derived neurotrophic factor in neurodegenerative diseases. Nature reviews Neurology, 5(6), 311‐322. 10.1038/nrneurol.2009.54 19498435

